# Role of methylene blue with fibrin glue in branchial cleft anomaly case report and review of literature

**DOI:** 10.1093/jscr/rjae385

**Published:** 2024-06-04

**Authors:** Ali M Alsudays, Homood Mohammad Almutairi, Abdulrahman N Almutairi, Mazyad M Alenezi, Wala S AlShiha

**Affiliations:** Department of Otolaryngology Head and Neck Surgery, Prince Sultan Military Medical City, Riyadh 11159, Saudi Arabia; Department of Otolaryngology Head and Neck Surgery, Prince Sultan Military Medical City, Riyadh 11159, Saudi Arabia; Department of Otolaryngology Head and Neck Surgery, King Saud University Medical City, Riyadh 11564, Saudi Arabia; Department of Otolaryngology Head and Neck Surgery, College of Medicine, Qassim University, Qassim, Buraydah 52375, Saudi Arabia; Division of Pediatric Otolaryngology, Prince Sultan Military Medical City, Riyadh 11159, Saudi Arabia

**Keywords:** case report, branchial cleft, branchial cyst, methylene blue, fibrin glue

## Abstract

We report a 3-year-old girl who presented to our clinic with a left-sided neck mass at the junction of the middle and lower thirds of the anterior border of the sternocleidomastoid with a slight tenderness. The patient was then diagnosed with a branchial cleft and was taken for surgical excision. Intraoperatively, we injected methylene blue with fibrin glue using an arterial catheter inside the tract, which facilitated the dissection of the tract.

## Introduction

Branchial congenital anomalies are considered to be the second most common congenital anomaly in the head and neck in children. Embryologically, they resulted from the incomplete obliteration of branchial pouches and clefts. Patients usually present with painless lateral neck swelling. Imaging with computed tomography (CT) and magnetic resonance imaging (MRI) will aid in the diagnosis and provide additional information regarding the extent and composition of the mass. Surgical resection of the mass is the mainstay in the management of branchial clefts.

## Case

A 3-year-old girl, medically free, presented to our otolaryngology head and neck surgery clinic with a very small left-sided neck mass associated with a yellowish discharge and pain at the mass site that was noticed at the age of 2 years by her mother. The family also noticed the presence of a small neck opening immediately after birth at the same site of this swelling. The patient has a history of recurrent tonsillitis, more than seven times per year over the past year. The mother denied any history of local erythema, fever, trauma, insect bites or other neck masses. In addition, she has bilateral preauricular sinuses that are asymptomatic on the right side, but there is a history of erythema and discharge on the left side. They also reported a positive family history of similar conditions.

On examination, there is a left-sided small punctum at the junction of the middle and lower third of the anterior border of the sternocleidomastoid with a very slight tenderness that became more prominent by tilting the head to the other side with yellowish discharge. There was no erythema, skin changes or lymphadenopathy. Also, there are bilateral preauricular sinuses with no signs of inflammation.

A CT scan showed a left-sided infected cystic lesion that is consistent with the preauricular sinus but did not show any cysts or tracts of the branchial cleft cyst (Fig. 5). Renal ultrasound showed normal findings and play audiogram showed normal hearing level. The patient consented for direct laryngobronchoscopy with excision of the branchial fistula, as well as preauricular sinuses excision and tonsillectomy.

### Intraoperative finding

**Figure 1:**
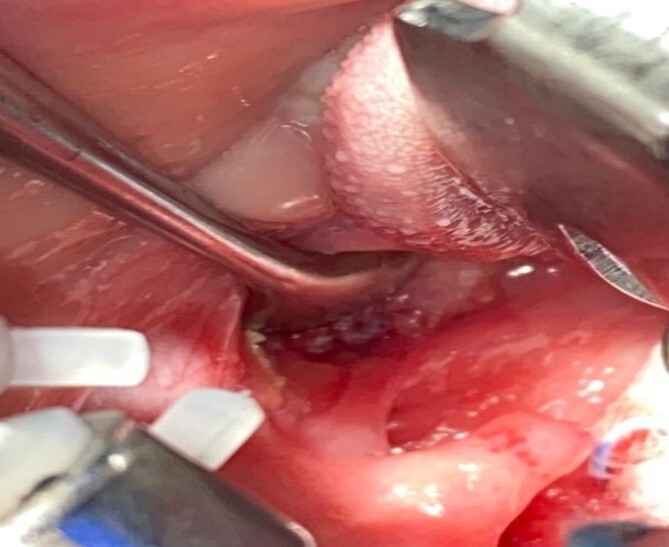
Left tonsil with clear intraoral fistula opening colored by methylene blue with fibrin before removal.

**Figure 2:**
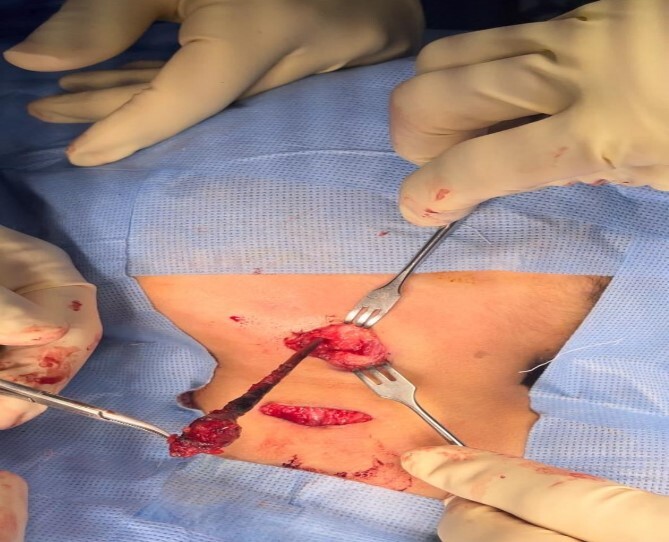
The branchial firm colored tract seen clearly after delivery it by upper step ladder incision. Notice the tract removed without excessive tissue removal around it.

**Figure 3:**
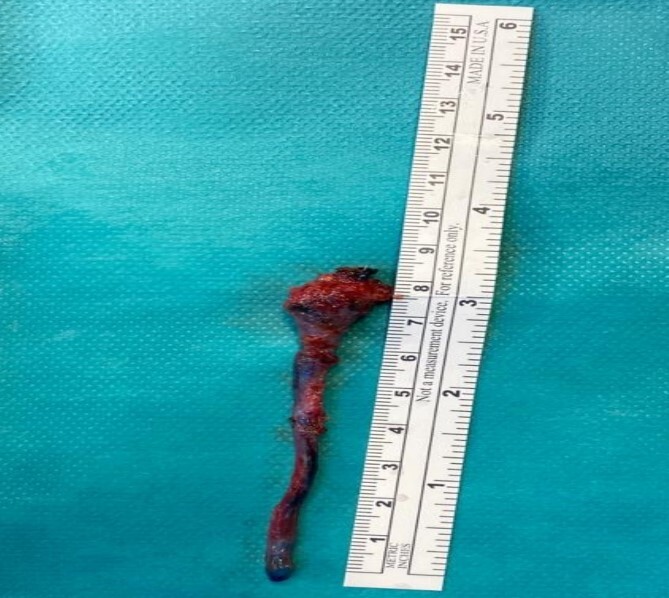
The complete neck tract after excision.

**Figure 4:**
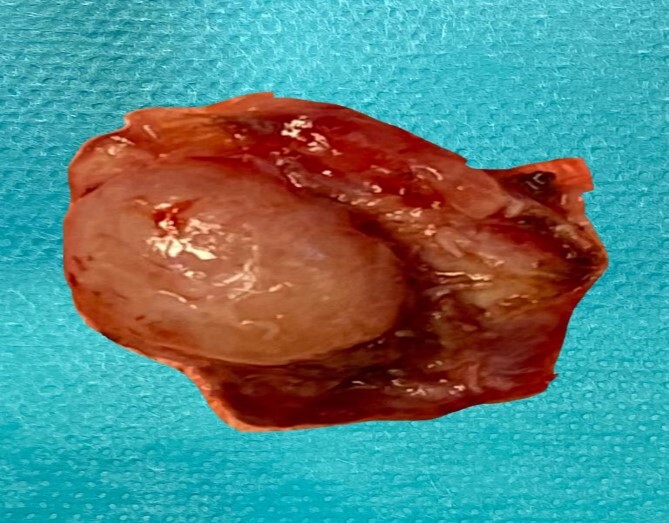
The left tonsill after removal and tract opening seen clearly on its surface.

After draping and sterilisation, the small left-sided neck punctum was identified, probing of the fistula tract by a lacrimal probe was done, followed by the insertion of an arterial catheter inside the fistula tract (Fig. 6). The amount of methylene blue added was 0.01 ml per 2 ml of fibrin glue, and a total of 4 ml of this mixture was injected. This mixture allows the glue to be dyed adequately and does not inhibit its solidification. The arterial catheter was totally inserted, about 7 cm in length, and injection of methylene blue with fibrin glue was performed using an arterial catheter inside the tract. An elliptical incision was made around the fistula opening, followed by dissection of the tract. The tract was obvious, firm and colored with methylene blue. The tract was easily dissected without the need to remove extra tissues around it. Another superior incision, a step ladder incision, was made as the tract was long. The tract followed to its entry into the tonsillar fossa, which was ligated. The internal carotid artery, internal jugular vein, hypoglossal nerve and glossopharyngeal nerve were all identified and preserved. Bilateral tonsillectomy was performed, and the tract was visualised within the left tonsil (Figs 1–4). The left tract at the tonsillar bed after the tonsillectomy was ligated and cauterised. Then bilateral auricular sinus excision is done. The patient was then seen after 3 months of surgery and was doing well.

**Figure 5:**
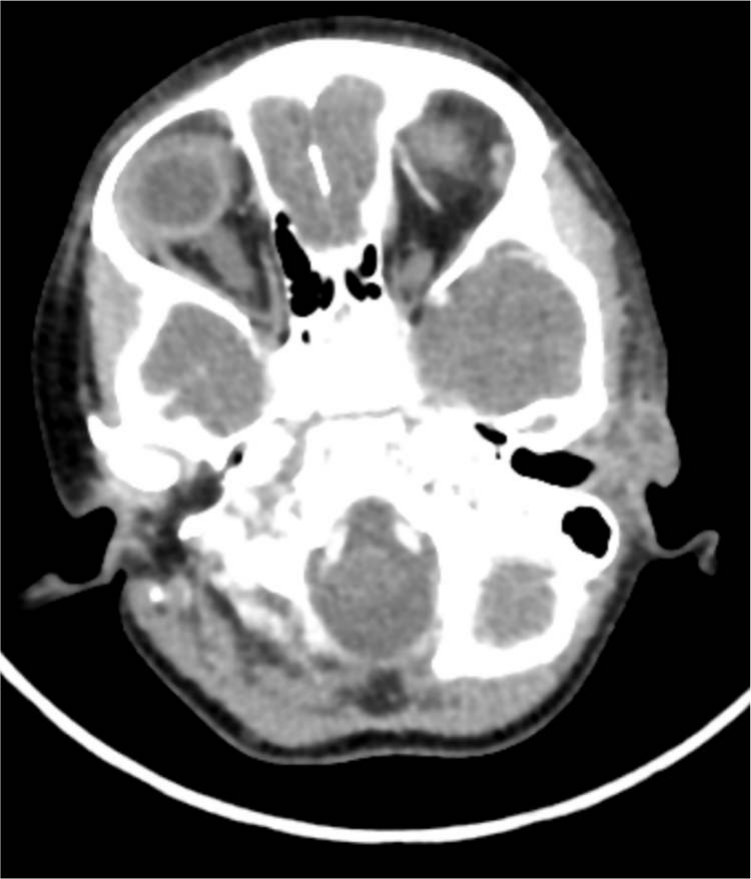
This is the axial cut CT that showed left-sided cystic lesion representing infected preauricular sinus. The CT did not show the second branchial cyst or tract that was found in clinical examination.

**Figure 6:**
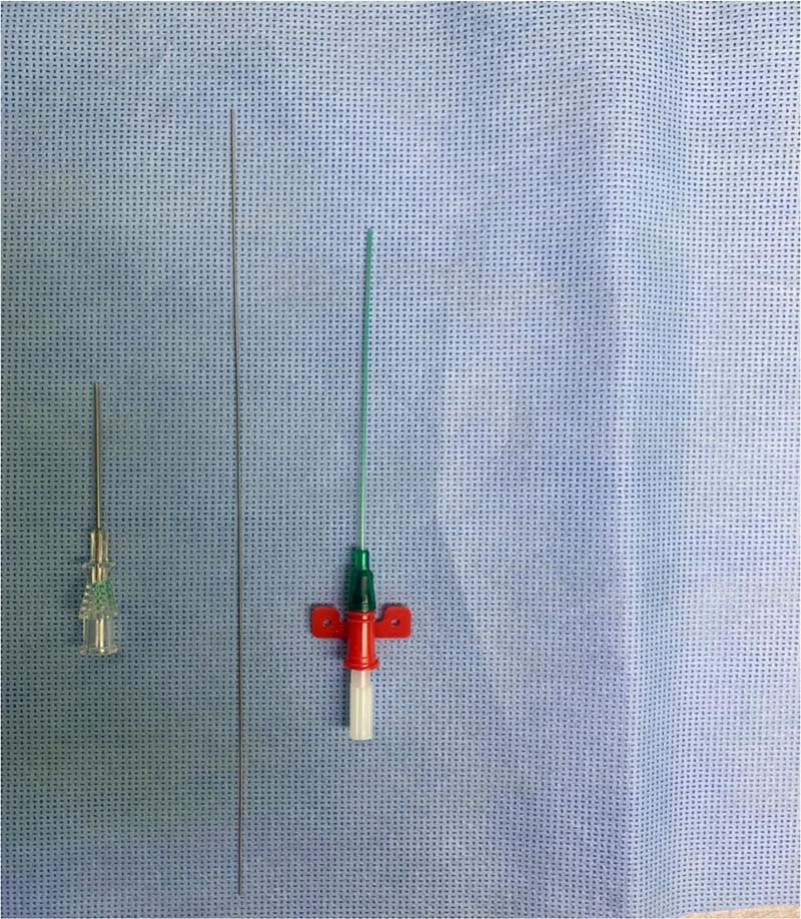
Arterial catheter that is used to inject methylene blue with fibrin.

### Review of literature and discussion

Branchial congenital anomalies are considered to be the second most common congenital anomaly in the head and neck in children. They account for about 30% of congenital neck anomalies. Embryologically, they resulted from the incomplete obliteration of branchial pouches and clefts. Patients with branchial clefts may present with a cyst, sinus, fistula or only a remnant of cartilage [1]. Branchial cysts are lined by stratified squamous epithelium resting on a complete or incomplete band of lymphoid tissue and have no external or internal openings. Branchial sinuses are tube-like lesions, with or without cystic lesions, that have an opening on the skin of the neck and end blindly on the deeper part of the neck. On the other hand, branchial fistulas resemble the sinuses, but they have an opening on the pharynx [1–3]. About 90% of branchial cleft anomaly cases are unilateral and occur on the right side of the neck. There are four types of branchial cleft anomalies; 8% of cases of branchial cleft anomalies are of the first branchial type, which is subdivided into Work types 1 and 2. Work type 1 shows a preauricular sinus that has a track medial and anterior to the external auditory canal, which contains ectoderm only. Work type 2 contains both mesoderm and ectoderm and presents at the mandible angle or within the submandibular region that has a close association with the facial nerve [1, 4, 5]. On the other hand, 90–95% of cases of branchial cleft anomalies are of the second type. The second branchial sinus/fistula opens externally in the lower neck along the anterior border of the sternocleidomastoid (SCM) muscle. The fistula tract extends upward and medial to the facial nerve above the hyoid bone and adjacent to the cranial nerves IX, XII and carotid sheet, then opens at the tonsillar fossa [1, 6]. The third branchial cleft anomalies are more commonly found on the left side in the lower portion of the anterior neck that run superficially to cranial nerve X and common carotid artery and to cranial nerve XII but deep to cranial nerve IX. Moreover, they open in the base of the pyriform sinus. The fourth branchial anomalies typically present as a low anterior neck mass located anterior to the sternocleidomastoid muscle. The tract of the fourth branchial cleft anomalies loops around cranial nerve XII, then runs posterior to the common carotid artery and thyroid, following the recurrent branch of cranial nerve X. It then passes through the cricothyroid membrane below the superior laryngeal nerve and drains into the apex of the pyriform sinus [5].

Branchial cleft anomalies are present at birth, but in many cases, they do not become evident until later in adolescence or childhood. Cases of branchial cysts in adulthood rarely occur, and in most cases, they are misdiagnosed with a metastatic nodal disease [7]. In children, the first manifestation of branchial cleft anomalies is a painless, fluctuant mass on one lateral side of the neck. Furthermore, a small opening in the skin on the side of the neck can be observed that usually drains fluid or mucus [8]. In case of an infection, the patient will feel pain, become feverish, and experience redness and swelling on the side of the neck. Fistulas are usually asymptomatic until the emergence of an infection [9]. In some cases, the swelling on the neck of the child is usually accompanied by an upper respiratory infection [10]. CT provides additional data with consideration of the extent and internal composition of the branchial cleft anomalies and their relation to neurovascular structure. MRI is required in some cases where preoperative assessment is needed [11].

Regarding management, surgery is usually considered the main method of treating branchial cleft anomalies, but it has some difficulties, especially in locating the position of the entire branchial cleft anomaly, with the risk of residual and subsequent recurrence. Intralesional usage of methylene blue is another way of treating branchial cleft anomalies, as it helps to identify the tract and know its extension. However, this method has its limitations, as it has a high rate of spreading into neighboring tissues. However, by adding fibrin glue to methylene blue, it is more likely to prevent the spread of methylene blue to other tissues. The use of fibrin glue dyed with methylene blue Intraoperatively for branchial cleft anomalies was first done by Piccioni et al. they approved this method when they did a retrospective single-center cohort study in 2016, at the Department of Pediatric Otolaryngology of the Spedali Civili of Brescia. They were applied to 17 patients suffering from branchial anomalies in Italy. After a mean follow-up of 47.8 months, all patients were free of the disease. The combination of methylene blue with fibrin glue forms a steady-dyed gel that inhibits the dye from dispersing. During surgery, the intralesional dose of dyed fibrin glue identifies the extent of the dissection without unnecessary dissection of adjacent areas. The use of methylene blue together with fibrin glue does not obstruct the conclusive histological analysis, making it an efficient and effective method in the treatment of branchial cysts. Worth mentioning are the possible complications or side-effects of using this mixture, including skin irritation and systemic hypersensitivity, the wrong injection into a blood vessel, which can cause hyperbilirubinemia, respiratory distress, pulmonary edema and bluish discoloration of tracheal secretions and urine [6, 10, 11].

## Conclusion

The mixture of methylene blue with fibrin glue helps in the resection of branchial cysts as it causes stiffness and demarcation of the tract, which helps during dissection. Also, with the amalgamation of fibrin glue, it is likely to prevent the spread of methylene blue to other tissues.

## Declarations

### Consent for publication

A written consent taken from the father of the child.

### Authors’ contributions

AMS, case writing and literature review.

H.M.M., case writing and literature review.

A.N.M., case writing and literature review.

M.M.E., participating in the surgery and reviewing the article.

W.S.S., participating in the surgery and reviewing the article.

### Conflict of interest statement

None declared.

## Funding

No funding received.

## Data Availability

The datasets used and/or analyzed during the current study are available from the corresponding author on reasonable request.

## References

[1] Shen LF , Zhou SH, Chen Q, et al. Second branchial cleft anomalies in children: a literature review. Pediatr Surg Int 2018;34:1251–6. 10.1007/s00383-018-4348-8. 30251021

[2] Chaouki A , Lyoubi M, Lahjaouj M, et al. Atypical first branchial cleft fistula: a case report. Int J Surg Case Rep 2021;78:159–61. 10.1016/j.ijscr.2020.12.007. 33352445 PMC7753191

[3] Howie AJ , Proops DW. The definition of branchial cysts, sinuses and fistulae. Clin Otolaryngol 1982;7:51–7. 10.1111/j.1365-2273.1982.tb01561.x. 7060284

[4] Work WP . Newer concepts of first branchial cleft defects. Laryngoscope 2015;125:520–32. 10.1002/lary.25202. 25677102

[5] Coste AH , Lofgren DH, Shermetaro C. Branchial Cleft Cyst. Treasure Island (FL): StatPearls Publishing; 2024 [cited 2024 Apr 2]. Available from: http://www.ncbi.nlm.nih.gov/books/NBK499914/. 29763089

[6] Pitak-Arnnop P , Subbalekha K, Sirintawat N, et al. Intraoperative injection of combined fibrin sealant and methylene blue dye for surgery of branchial cleft cysts: a case report. J Stomatol Oral Maxillofac Surg 2019;120:378–82. 10.1016/j.jormas.2019.02.013. 30797901

[7] Arshad M , Ashafaq U, Aslam M. Branchial cleft cyst. Prof Med J 2019 Mar 10 [cited 2024 Apr 2];26:4. Available from: http://theprofesional.com/index.php/tpmj/article/view/3263.

[8] Rambabu V , Kishore J, Dinesh Kumar Reddy N, et al. A clinico-pathological study of neck swellings excluding thyroid. JEBMH 2015;2:6046–50.

[9] Kim SC , Kim JH, Won JK, et al. Asymptomatic intrathyroidal pyriform sinus fistula mimicking thyroid cancer: a case report and literature review. Medicine (Baltimore) 2018;97:2–3, e0488. 10.1097/MD.0000000000010488. 29668629 PMC5916699

[10] Piccioni M , Bottazzoli M, Nassif N, et al. Intraoperative use of fibrin glue dyed with methylene blue in surgery for branchial cleft anomalies: Piccioni et al.: methylene blue and fibrin glue. Laryngoscope 2016;126:2147–50. 10.1002/lary.25833. 26927898

[11] Mittal MK , Malik A, Sureka B, et al. Cystic masses of neck: a pictorial review. Indian J Radiol Imaging 2012;22:334–43. 10.4103/0971-3026.111488. 23833426 PMC3698897

